# Synaptoimmunology - roles in health and disease

**DOI:** 10.1186/s13041-017-0308-9

**Published:** 2017-06-20

**Authors:** Robert Nisticò, Eric Salter, Celine Nicolas, Marco Feligioni, Dalila Mango, Zuner A. Bortolotto, Pierre Gressens, Graham L. Collingridge, Stephane Peineau

**Affiliations:** 1 Department of Biology, University of Rome Tor Vergata, 00133 Rome, Italy; 2 Pharmacology of Synaptic Disease Lab, European Brain Research Institute, 00143 Rome, Italy; 3 Department of Physiology, University of Toronto, and Lunenfeld-Tanenbaum Research Institute, Mount Sinai Hospital, Toronto, ON Canada; 4 Centre for Synaptic Plasticity, School of Physiology, Pharmacology & Neuroscience, University of Bristol, Bristol, UK; 5 PROTECT, INSERM, Université Paris Diderot, Sorbonne Paris Cité, Paris, France; 6 Centre for the Developing Brain, King’s College, St Thomas’ Campus, London, UK; 7 INSERM-ERi 24 (GRAP), Centre Universitaire de Recherche en Santé, Université de Picardie Jules Verne, Amiens, France

**Keywords:** Nervous system, Immune system, Synaptic plasticity, Neuroinflammation, Microglia

## Abstract

Mounting evidence suggests that the nervous and immune systems are intricately linked. Many proteins first identified in the immune system have since been detected at synapses, playing different roles in normal and pathological situations. In addition, novel immunological functions are emerging for proteins typically expressed at synapses. Under normal conditions, release of inflammatory mediators generally represents an adaptive and regulated response of the brain to immune signals. On the other hand, when immune challenge becomes prolonged and/or uncontrolled, the consequent inflammatory response leads to maladaptive synaptic plasticity and brain disorders. In this review, we will first provide a summary of the cell signaling pathways in neurons and immune cells. We will then examine how immunological mechanisms might influence synaptic function, and in particular synaptic plasticity, in the healthy and pathological CNS. A better understanding of neuro-immune system interactions in brain circuitries relevant to neuropsychiatric and neurological disorders should provide specific biomarkers to measure the status of the neuroimmunological response and help design novel neuroimmune-targeted therapeutics.

## Introduction

Following a pathogenic insult to the brain, most central nervous system (CNS) cells, as well as some peripheral immune cells, participate to the genesis of a central inflammation known as neuroinflammation. This process consists of complex biochemical cascades that serve as a protective mechanism to eliminate the initial cause of cell injury and promote recovery. For many years it was thought that the immune system within the CNS served exclusively a reactive role following insults to the brain. However, recent evidence suggests that the brain and immune system are intimately linked and engage in significant cross-talk under physiological, not just pathological, conditions to preserve homeostasis. Indeed, several proteins first detected in the immune system have been found also in the healthy uninfected nervous system, where they are having pleiotropic functions. Conversely, proteins first described in the nervous system have since been associated with immunological functions [1]. These factors influence numerous physiological functions including neurite outgrowth, neurogenesis, neuronal survival, synaptic pruning, synaptic transmission and synaptic plasticity [2]. We have termed the interplay between immune modulators and synaptic function, synaptoimmunology.

In this review we first provide a summary of the mechanisms of synaptic transmission/plasticity and immune cell signaling pathways. Then, we discuss how these pathways converge and thus play a role in numerous physiological functions of synapses, with an emphasis on synaptic plasticity. Finally, we describe how synaptoimmunology is involved in a variety of different brain disorders.

### Synaptic communication

Synapses are the main points of rapid communication between neurons (and in some cases between neurons and astrocytes or microglia), through the use of chemical neurotransmitters. This communication is subject to alteration, a phenomenon known as synaptic plasticity: synaptic strength can be enhanced (potentiation, P) or reduced (depression, D). The duration of the alteration could be transient (on the order of s and min) or more stable (h to years) and is defined as short term (ST) or long term (LT) respectively. Both parameters define the type of plasticity occurring at the synapses: LTP, LTD, STP, STD. Mechanistically, synaptic plasticity modulates either the function of membrane proteins (gain or loss) or their availability (endocytosis, exocytosis, degradation, synthesis). All these events are under the control of multiple intracellular signaling pathways [3–5].

Neurons are also able to communicate through volume communication, mainly via diffusing peptide molecules (e.g., neuropeptides, cytokines, growth factors). This communication can arise between neurons but also between the different cell types of the CNS (neurons, astrocyte, microglia, oligodendrocytes, endothelial cells, immune cell, etc.). These peptide molecules are more slowly degraded or captured compared to the smaller sized transmitters and can thus diffuse far from their release site. Once they reach their target (GPCR, kinase receptor, etc.), intracellular signaling pathways are activated. On neurons, these receptors can be located at, or in the vicinity of, synapses, where they can directly modulate synaptic functions [6–8].

### Cell signaling at synapses

The activation of intracellular signaling pathways at synapses, as a response to synaptic events or diffusing molecules, can lead to the modification of the local synaptic strength and also a more generalized alteration in neuronal function that often involves changes in gene expression. Consequences of this signaling can therefore be restricted to a local action mode within, or in the vicinity of, the stimulated synapse, or can involve interactions between the soma and synapses.

**Fig. 1:**
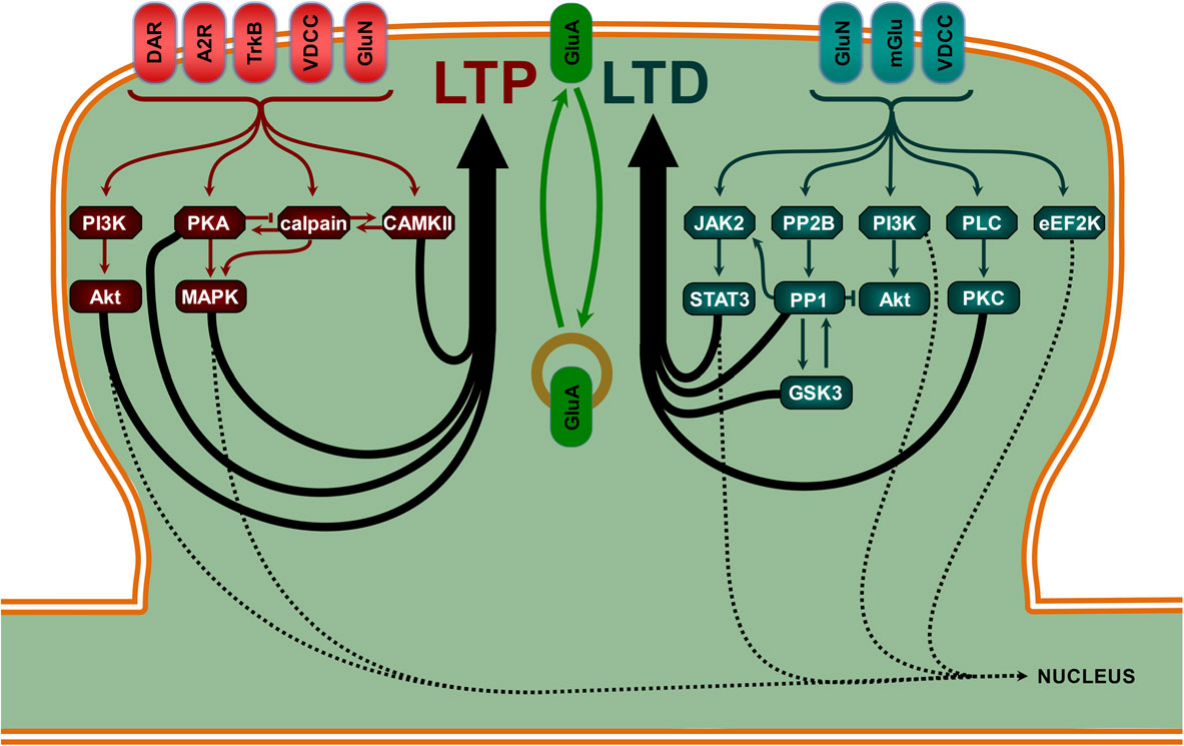
Some of the main signaling pathways in LTP and LTD. LTP involves (at different synapses) several type of receptors which include NMDA receptor (GluN), voltage dependent calcium channel (VDCC), neurotrophin receptor (trkB), adenosine 2 receptor (A2R) or dopamine receptor (DAR). These receptors activate intracellular signaling pathways with local and/or somatic effects, such as phosphoinositide-3 kinase (PI3K)/Akt, protein kinase A (PKA)/ mitogen activated protein kinases (MAPK), calpain/ striatal enriched protein tyrosine phosphatase (STEP) and calcium calmodulin kinase II (CaMKII) pathways. LTD can be triggered by the activation of, for example, GluN, VDCC and metabotropic glutamate receptors (mGlu), depending of the form of LTD. Calcineurin (PP2B)/protein phosphatase 1 (PP1) associated to Janus kinase 2 (JAK2)/ signal transducers and activators of transcription 3 (STAT3), PI3K/Akt and glycogen synthase kinase 3 (GSK3) are mainly required for GluN dependent LTD whereas mGlu dependent LTD activates mainly phospholipase C (PLC)/Protein Kinase C (PKC) and eukaryotic elongation factor 2 kinase (eEF2K) signaling pathways. Sequence of activation of these pathways and inter-regulation between them are two key features to obtain synaptic plasticity events

Synaptic strength is modified by synaptic plasticity events. During LTP, some kinases cascades, such as CaMKII, have a strict local action as they are involved in synaptic cytoskeleton remodeling, AMPAR trafficking and/or local protein synthesis. The PKA cascade, however, can both affect glutamatergic receptor properties locally as well as regulate somatic transcription and translation [9–11]. Considerable cross-talk exists between these different cascades. A similar scheme also exists for LTD: PP1 or PLC cascades modify AMPAR trafficking and internalization, whereas JAK/STAT, PI3K and eEF2K have both local and somatic roles (Fig. 1) [3, 12].

Whilst most forms of synaptic plasticity are induced by activation of glutamatergic receptors, synapses express numerous other receptors including neuropeptide receptors, cytokine receptors, growth factor receptors, which represent potentially hundreds of receptors able to detect circulating molecules. Interestingly, many of these receptors engage the same signaling pathways as those involved in synaptic plasticity. This potentially enables many ways in which neuropeptides, and other neuromodulators, can affect synaptic plasticity and other synaptic functions (Fig. 2).

**Figu. 2:**
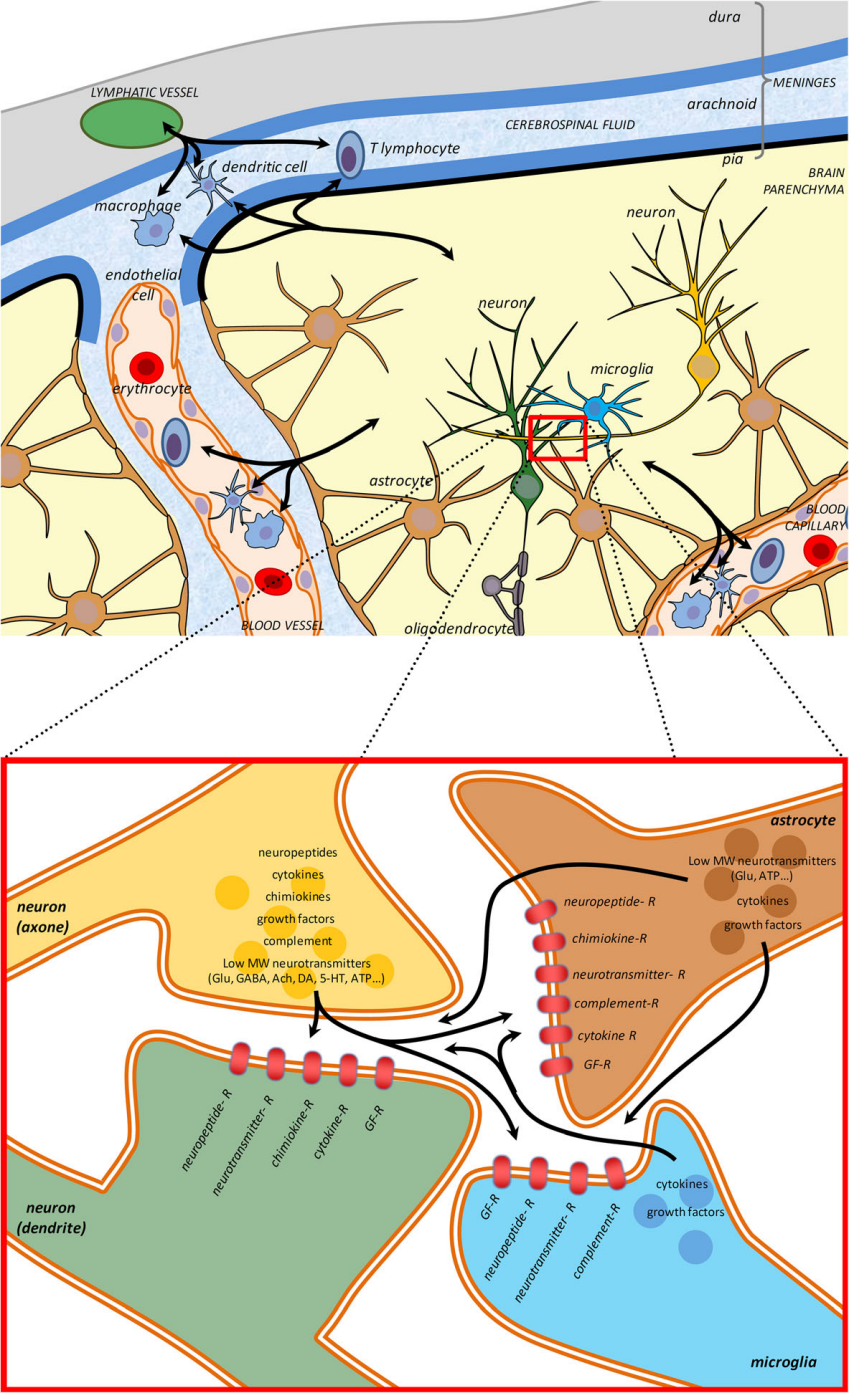
A schematic of a synapse showing pre, post elements, astrocytes and microglia. Brain and immune cells undergo a dynamic dialog. Peripheral immune cells, such as T-lymphocytes, macrophages and dendritic cells, coming from the cerebrospinal fluid or carried by blood vessels penetrate the brain through the blood brain barrier. They either have a surveilling activity or are attracted by the chemokines released by injured tissues. Microglia, the brain resident immune cells, perform a constant surveilling activity and are in particular attracted by synapse activity, locus of an intense interplay between neurons and glial cells. Many neurotransmitters circulate between these cell types resulting in the modulation of the synaptic functions. Increasing evidence suggest that molecules and signaling pathways first discovered for the immune system takes an important place in the physiological functioning of the synapse. Growth factor receptor (GF-R); Glutamate (Glu); Gamma Amino Butyric Acid (GABA); Acetylcholine (Ach); Dopamine (DA), Serotonin (5-HT), Adenosine tri-phosphate (ATP). (top scheme, cerebral structure inspired from [37])

### Immune system signaling

The immune system acts to defend against and restore homeostasis following the invasion of foreign pathogens and tissue damage. This is achieved by the two arms of the immune system, the innate and adaptive systems, the former being a more rapid, nonspecific response while the latter is slower and specific to a particular antigen. Pattern Recognition Receptors (PRR) on the surface immune cells detect damage associated molecular patterns (DAMPs, like heat shock proteins, ATP, cell fragments or mRNAs) released following tissue damage as well as pathogen associated molecular patterns (PAMPs, such as lipopolysaccharide) found on the surface of microbes. Activation of Toll-like receptors (TLRs), in cooperation with other PRRs, leads to the expression and release of cytokines and other inflammatory molecules (TNF, IL-1β, IL-6, NO, etc.), via activation mainly of NFƙB, MAPK and caspase-1 [13, 14]. The factors released attract other immune cells and activate a variety of specific receptors. Depending on the receptor, different signaling pathways can be activated, all leading to the modulation of genes regulating cell proliferation or apoptosis. Cytokine receptors activate mainly the JAK/STAT pathway [15, 16] which can either regulate the expression of apoptotic molecules such as Bcl-xL or cell proliferation, depending on the isoforms activated. Chemokines can activate GPCRs which control the PKA/CREB signaling pathway, while growth factors, for example, can activate PI3K/Akt and the MEK/ERK pathway, via tyrosine receptors, to regulate gene expression [17]. There is considerable cross-talk between these different pathways during the inflammation and healing process.

### The immune system in the CNS

Seminal experiments in the 1920s demonstrated that, unlike peripheral tissue, engraftment of tumors into the brain parenchyma did not elicit an immune response and thus the tissue graft was not rejected [18, 19]. This gave rise to the idea that the brain is an immune-privileged organ; an idea which still holds though has been greatly refined. Immune-privilege does not refer to an absolute but rather relative state, and the level of immune-privilege differs between compartments of the brain [20, 21].

In the healthy brain, the subarachnoid space, cerebrospinal fluid and vasculature contain circulating leukocytes including dendritic cells, macrophages and T cells, however the entry of these cells into the brain parenchyma is highly restricted and regulated [21]. The brain parenchyma instead contains the tissue-resident macrophages known as microglia, which are a self-renewing population derived from yolk-sac myeloid precursor cells which invade the CNS between E8.5 and E9.5 in mice [22]. Two groundbreaking studies [23, 24] examined microglia dynamics in the healthy, uninjured adult brain using in vivo imaging and unexpectedly found that microglia processes are continuously surveying the brain parenchyma and are the most morphologically plastic cells in the CNS. Additionally, microglia processes were found to contact synapses and this interaction can be modified by neuronal activity [25, 26]. These studies shifted the view of microglia in the healthy brain from quiescence to active surveillance, and gave rise to the notion that microglia play a role in synaptic physiology. From a surveillance mode, various stimuli can cause the microglia activation which might lead to changes in morphology (for example, from ramified to amoeboid), the release of cytotoxic or neuroprotective factors (such as cytokines and growth factors), alterations in gene and surface receptor expression, and phagocytosis of tissue debris or pathogens [27–29].

Astrocytes are the main resident glial CNS cell population and are the second main source of brain cytokines. Following brain injury, astrocytes are activated and form a reactive astrogliosis, a process important for isolating the injured area and protect the adjacent cells but which is deleterious for neuronal axonal regeneration [30, 31]. Astrocytes also form the glia limitans surrounding the vasculature of the CNS and control the entry of peripheral immune cells into the brain parenchyma [21].

Additionally, injured neurons release factors such as cytokines and neurotransmitters that recruit and activate the other CNS cells involved in neuroinflammation. For example, microglia can be activated by cytokines, by the detection of cellular damages [13] and by neurotransmitters released during injury [32]. Activated microglia release a large array of inflammatory mediators leading also to the recruitment of peripheral macrophages, dendritic cells and T lymphocytes [14, 33–36] directly from meningeal lymphatic vessels or blood vessels [37, 38]. Astrocytes and adjacent neurons are also activated in parallel to this cascade leading to further factor release [39] (Fig. 2).

Most of the dialog between these different cell types is due to factors released in the extracellular medium (volume communication) though direct contacts are involved in some key steps. Neurons express most of the receptors associated with the released factors during neuroinflammation and are sensitive to the gazotransmitters or reactive oxygen species released by inflammatory cells. It remains to be determined how the subcellular localization of these receptors is important to modify synaptic activity and whether they have to be present at the modified synapse or if they can affect it at distance.

## Synaptoimmunological mechanisms involved in physiological processes

Initially believed to play a role only during inflammation processes, increasing evidence suggest that immune signals are essential components of normal synaptic functioning, including roles in synaptic plasticity and stability [2].

### Synaptic plasticity

Most synaptic plasticity occurs at glutamatergic synapses, where transmission is mediated by ionotropic receptors (GluN, GluA and GluK) and metabotropic receptors (mGlu). Transporters, expressed on neurons and astrocytes, limit the activation of glutamate receptors. All these receptors are subject to many post-translational modifications, amongst which two of the most important are protein/protein interactions and phosphorylation.

One main role of cytokines in synaptic transmission is their ability to modulate the induction of synaptic plasticity. IL-1β, IL-2, IL-6, IL-8, IL-18, IFNα, IFNγ and TNFα have all been shown to be able to inhibit LTP and to induce changes in hippocampal dependent learning and memory tasks [40–42]. LTD can also be inhibited either directly by cytokines like IL-1β or during the inflammation process [40, 43]. Conversely, under physiological conditions, activation of the JAK2/STAT3 pathway is required for the induction of GluN-dependent LTD in the hippocampus [12]. Further the induction of LTP increases the expression of IL-6 which acts in a negative feedback manner to limit the magnitude of potentiation [44, 45]. These results demonstrate that cytokine signaling not only acts in a metaplastic manner to modulate bidirectional changes in synaptic efficacy, but is also part of the physiological mechanism.

The IL-1β receptor has been shown to physically interact with GluN receptors enabling the rapid regulation of GluN activity, via Src-dependent phosphorylation events [46]. The IL-1β receptor can also decrease GluA surface expression [47]. TNFα can promote GluA dependent activity in hippocampal neuron and may induce GluA internalization in striatal GABAergic neurons (reviewed in [48]).

The mechanisms by which cytokines or inflammation alter synaptic function is complex as microglia by themselves can directly facilitate synaptic strength independently of any changes in synaptic activity. Once activated, they can, for example, induce STP via IL-1β dependent mechanisms [49] or LTD in pathological context [50].

In addition to cytokines, major histocompatibility complex (MHC) class I molecules also play a role in modulating the induction of synaptic plasticity. MHC class I molecules are a group of proteins which, within the immune system, translocate cytosolic peptides generated by proteasome-mediated degradation to the cell surface for recognition and subsequent cell elimination by cytotoxic T cells [51]. MHC class I molecules have also been found to be expressed by neurons in the CNS and localize to synapses, axon terminals and dendrites [52–56]. In the dorsal lateral geniculate nucleus (dLGN), the MHC class I molecule H2-D^b^ was found to be necessary in limiting the synaptic incorporation of calcium-permeable AMPARs and thus permit the induction of LTD [57]. In the hippocampus, β_2_M−/− TAP−/− mice (which lack cell surface expression of MHC class I molecules) have a shift in LTP threshold in area CA1, such that low-frequency stimulation, which usually induces LTD, instead causes LTP, and LTP induced by high-frequency stimulation is larger in magnitude [54, 58]. Finally, MHC class I acts as a negative regulator of synapse density in both the cortex and hippocampal area CA3 [59, 60], which, in area CA3, is the result of a MHC class I-insulin receptor complex which constrains basal insulin receptor signaling [60].

Finally, the complement system, which is part of the innate immune system, and in particular the complement component C3, could also play a direct role by modulating the efficiency of glutamatergic synaptic transmission in the absence of any inflammation process, by a mechanism not yet explored [61], but that could involve synaptic stripping [62], a process initially defined as the removal of dysfunctional synapses by activated microglia [63].

### Structural plasticity

Organisms are born with an excess number of synapses throughout the CNS, and during development superfluous connections are removed in an experience-dependent manner, a process known as synaptic pruning [64]. Within the CNS synaptic pruning has been extensively studied in the visual system, specifically the LGN and the striate cortex. Initially, LGN neurons receive inputs from multiple retinal ganglion cells (RGCs), however during postnatal development inputs are selectively removed such that eventually each LGN neuron only receives input from one or two RGCs [65]. Additionally, monocular deprivation (MD) during the developmental critical period can lead to reduced responsiveness to the deprived eye in the LGN and striate cortex as deprived eye inputs are weakened [66]. Stevens and colleagues [67] found that the classic complement cascade mediated the elimination of RGC inputs onto LGN neurons via microglia phagocytosis early in postnatal development (P5). A subsequent study found that complement-mediated synaptic pruning in the LGN is regulated by neuronal activity, as inhibiting activity in one eye with TTX increased microglia engulfment of that eye’s inputs, while the converse occurred when RGC activity in one eye was stimulated with forskolin [68]. The developing visual system model has also revealed the involvement of other immune pathways in synaptic pruning including purinergic signaling with microglia via the P2Y12 receptor [69] and MHC class I proteins [57].

In the developing hippocampus it has been demonstrated that puncta of the postsynaptic protein PSD-95 are contained within microglia, providing evidence of microglia-mediated synaptic pruning. Further, knocking out the microglia-specific fractalkine receptor CX3CR1 resulted in an increased spine density in neonatal mice [70]. It was also found that CX3CR1 KO mice had impaired synapse maturation [70, 71], assayed by measuring the number of release sites per neuron-neuron connection, and thus the authors proposed that that synapse elimination allows for subsequent strengthening of remaining synapses [71]. However, CX3CR1 KO mice were also found to have enhanced IL-1β levels which resulted in a specific impairment of LTP [72], suggesting that the effects of deficient synaptic pruning and synapse maturation observed in CX3CR1 KO mice may not be directly linked.

It has been proposed that weakened synapses are subsequently ‘tagged’ with complement proteins to induce microglia phagocytosis [73]. Synapse elimination has been found to occur in the hippocampus following both mGlu- and GluN-dependent LTD [74–79]. Thus, future studies should seek to directly examine whether there is an interaction between synaptic depression and microglia phagocytosis.

In the adult brain, activation of microglia leads to the displacement of inhibitory synapses from the soma of neurons and is neuroprotective by a mechanism involving GluN activation [80–82]. Synaptic stripping by microglia participates in network remodeling but its exact role in pathology remains to be fully demonstrated [83].

Synaptic pruning can also occur independently of any physical interaction with glia. This mechanism has been well described for neurons with axon injury and occurs concomitantly with glial cell activation. Astrocytes as well as microglia release numerous factors (cytokines, chemokines, thrombospondins, etc.) that directly influence synapse integrity [84, 85].

Though research has mainly focused on microglia in relation to synapse elimination, recent advances have shed light on the role microglia also play in spine formation. The generation of CX3CR1-CreER mice allowed Parkhurst and colleagues [86] to conditionally deplete microglia or knockout microglial BDNF. Subsequent in vivo two photon imaging revealed that these manipulations impaired spine formation in the motor cortex following motor learning. Furthermore, in vivo two photon imaging of the somatosensory cortex of developing mice revealed that microglia contact of dendrites frequently led to spine filipodia formation [87]. Therefore, it appears that microglia can cause bidirectional modifications of dendrite spine structure but the underlying mechanisms (see also the review of Kettenman et al. [63]) involved in this process need more investigation by future studies.

### Synaptic scaling

At glutamatergic synapses, when extended periods of elevated or depressed neuronal activity occur, homeostatic mechanisms can be activated. They alter strength across all synapses to return activity to an optimal range, a process known as synaptic scaling [88]. Chronic (24–48 h) blockade of neuronal action potential firing or glutamatergic synaptic transmission results in a large increase in mEPSC amplitude (a putative measure of post-synaptic sensitivity to glutamate) as well as the number of surface AMPARs [89]. Stellwagen and Malenka [90] found that TNFα is both necessary and sufficient for scaling up of post-synaptic AMPARs. Interestingly, although both neurons and glia are capable of producing TNFα, the authors found that it is the glia-released TNFα that is critical for scaling up of synapses. A subsequent study found that β3 integrins are also necessary for scaling up of synapses, and that TNFα application increases the surface expression of β3 integrin [91]. Further, the authors found that levels of β3 integrin control surface AMPARs and thus mEPSC amplitude, suggesting a model in which glial cells, in response to reduce network activity, release TNFα leading to an increase in β3 integrin surface expression and subsequent accumulation of AMPARs at the synapse [92].

To address the physiological significance of TNFα-meditated synaptic scaling, Kaneko and colleagues [93] examined visual system. In the striate cortex, similar to the hippocampus, LTP was normal in TNFα−/− mice, however scaling up of AMPAR-mediated mEPSCs was absent. In vivo, TNFα−/− mice had impaired ocular dominance plasticity following MD, specifically having a complete deficit in the increase of non-deprived eye cortical response, despite a normal decrease in the cortical response for the deprived eye. Thus, synaptoimmunological factors are critical for both phases of ocular dominance plasticity, with the complement cascade mediating the loss of cortical responsiveness to the deprived eye, while TNFα mediates the compensatory, homeostatic increase in non-deprived eye cortical responsiveness.

## Synaptoimmunological mechanisms involved in acute brain disorders

Causes of brain disorders associated with neuroinflammation and synaptic function alteration are numerous. Some disorders appear following a brief episode associated an infection, whereas others have either undefined causes or genetic origins. The mechanisms involved in synaptic function alteration are dependent of the nature of the cause.

### Acute infection

Following a systemic or a direct brain infection, an immunological response is triggered and coordinated by the brain and the immune system. In general acute and long-term infections of the brain and spinal cord are produced mostly by traumatic injury, parasites, intoxication and systemic infectious diseases caused by viruses, bacteria, fungi and parasites, which penetrate the central nervous system. A common and potentially life-threatening form of generalized inflammatory response is sepsis, which is characterized by an overreaction of the immune system. The pathophysiology of the sepsis is highly complex and affects all types of brain cells and brain functions (for a comprehensive review of sepsis and brain dysfunctions see [94]).

Brain viral infections (e.g., by influenza, HIV, Herpes, West Nile virus (WNV) has been reported to both directly and indirectly (by promoting neuroinflammation) affect synaptic functions, leading to cognitive impairment [95–97]. For example, a recent study found that synapse loss in a mouse model of WNV infection is driven by the activation of the classical complement cascade in the hippocampus [62]. Further, various viral proteins reduce voltage dependent calcium channel [98] or GluA function [99]. Interestingly some viral effects require NMDAR activation or alteration through PKA and PKC dependent mechanisms (for a review see [96]). Additionally, certain viral dependent synapse impairments are also explained by the ability of viruses to indirectly induce the expression or interfere with the function of proteins associated with synaptic impairment like Aβ or APP [100].

### Acute brain injuries without pathogen

Acute brain injuries are often associated with an inflammation response in the absence of any pathogen, a mechanism termed sterile inflammation, which can be caused by multiple different events such as mechanical trauma, ischemia, stress, alcohol etc.. Three of the main pathologies arising from these sterile injuries are Traumatic Brain Injuries (TBI), epilepsy and stroke, pathologies which share common cell death mechanisms [101]. This inflammation has been associated both with the worsening of the pathologies and with the repair phase [101–104], but the mechanisms involved in the alteration of synapse functions could be pathology-specific.

Synapses, by their intrinsic complex architecture including PSD and adhesion molecules, contribute largely to the diffusion of mechanical trauma during TBI [105] suggesting that inflammation dependent alteration of synapse integrity may be directly involved in the severity of the pathology.

Epilepsy and inflammation are strongly linked (reviewed in [106]). Synapse elimination, sprouting and changes in synaptic strength are key features of this pathology. Inflammation impacts epilepsy directly by modulating synaptic activity via altered protein expression through the activation of the NFκB pathway or by altering synaptic channel activity via phosphorylation cascades [107]. Computational network modeling has also predicted that TNFα release by glia following inflammation can lead to epileptogenesis through scaling up of synapses [108].

The effect of sterile inflammation on synaptic function may also depend of changes in neuronal environment during pathology. Thus TNFα enhances LTP in the context of ischemia via a p38 MAPK dependent mechanism, whereas it blocks LTP in the physiological context [109]. The alteration of glutamatergic transmission disappears when inflammation resolved [40].

Another prominent cause of brain inflammation is alcohol abuse, as illustrated by the phenomenon of “binge drinking” [110]. It is well documented that alcohol directly affects glutamate receptors (GluRs) and other families of receptors [111–117] as well as synaptic plasticity [118]. Critically several studies in humans [113, 119, 120] and animals [121–124] have provided strong evidence that the effect of alcohol abuse on GluRs harms brain development, synaptic refinement and impairs cognitive functions.

## Synaptoimmunological dysregulation in neurodegenerative/autoimmune brain disorders

In recent years, the classical dichotomy between inflammation and neurodegeneration has been challenged by evidence suggesting that both aspects are interconnected both in neurodegenerative diseases, including Alzheimer’s disease (AD) and Parkinson’s disease (PD), and in traditional neuroinflammatory disorders, such as multiple sclerosis (MS) [125, 126]. Growing experimental evidence suggests that synapses may be the locus for abnormalities underlying these diseases. Indeed, perturbations in the induction, maintenance or reversal of LTP and LTD are a common thread in the different brain disease models [127, 128], as well as in human pathologies associated with inflammation [129]. However, there are disease-specific mechanisms of how synapse structure and function are precisely affected in each disorder. It is thus reasonable to postulate that the combination of abnormal expression of immune mediators along with other disease-specific features might contribute to the distinct etiopathogenesis of different conditions.

### Multiple sclerosis/EAE

Multiple sclerosis (MS), especially its relapsing-remitting form, is a complex immune-mediated disease [130]. The neuroinflammatory milieu that typically characterizes MS profoundly impacts the capability of neuronal systems to express normal plasticity, possibly leading to a state of decreased homeostatic reserve with negative consequences on cognitive performances. Inflammation-induced synaptic dysfunction appears in the very early phases of MS patients and in the experimental autoimmune encephalomyelitis (EAE), a well-established mouse model of multiple sclerosis. Accordingly, it was recently shown that intermittent (iTBS) or continuous theta burst stimulations (cTBS), delivered through a transcranial magnetic stimulation (TMS) device, modulate the expression of cortical plasticity in the acute inflammatory phases of MS patients. In general, LTP was always favored over LTD in response to repetitive synaptic activation in MS brains, and this effect was directly correlated with IL-1β levels in the CSF [129]. Similar results were also observed in hippocampal slices from EAE mouse, in which the facilitation of CA1-LTP was also mediated via enhanced IL-1β released from CD3+ T lymphocyte infiltrates or activated microglia, clearly detectable in the EAE hippocampus [43, 131]. Remarkably, preventive or pharmacological strategies restraining pro-inflammatory cytokines and oxidative stress were able to rescue synaptic alterations in the EAE model [132, 133].

### Alzheimer’s disease

**Fig. 3:**
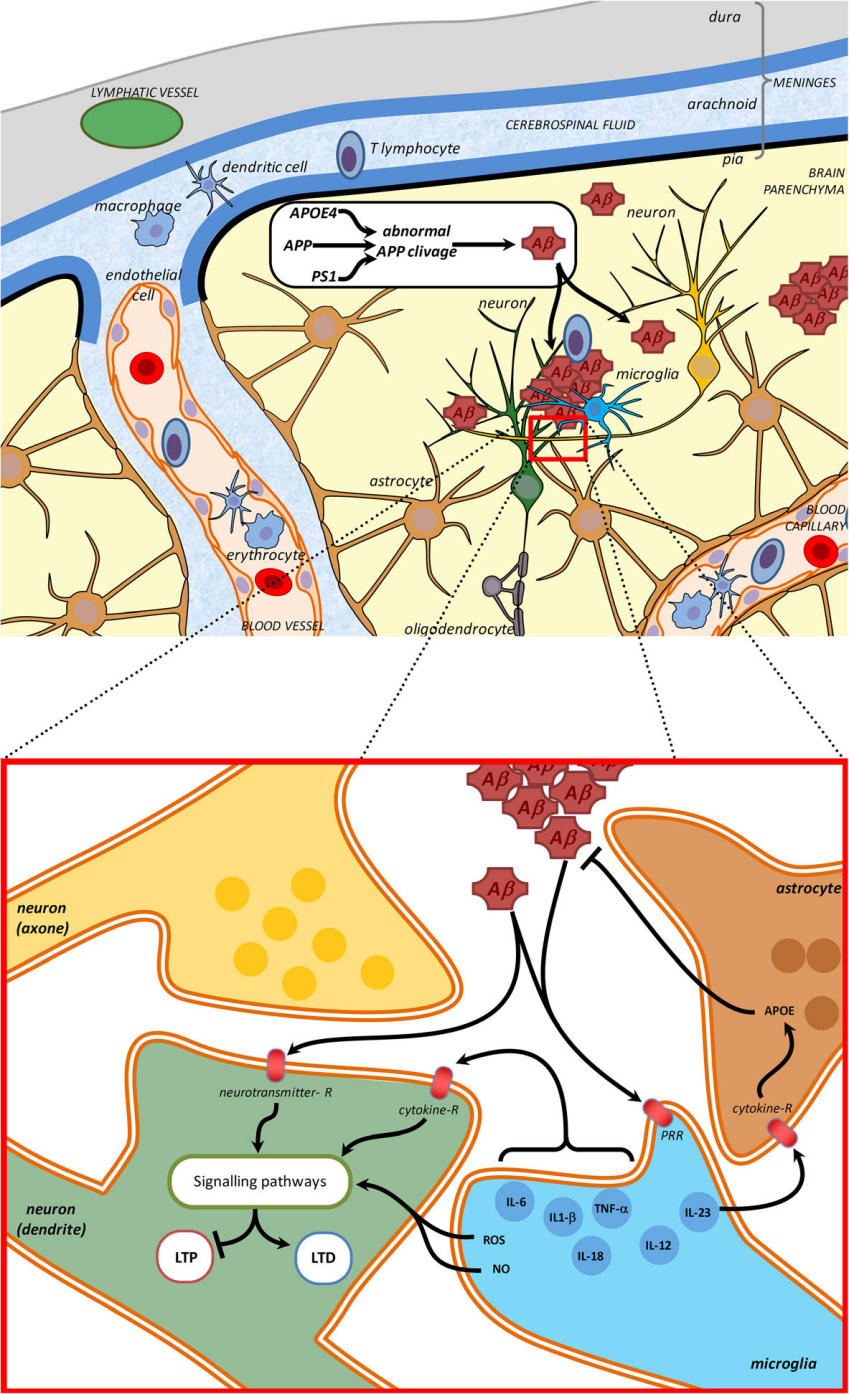
Signaling pathways in inflammation / immune response and how genetic risk factors for (e.g. AD) may impact via these pathways. Oligomeric or aggregate of amyloid beta peptides (Aβ), as occurring during Alzheimer’s disease, are detected by pattern recognition receptor (PRR) like Toll like receptors. In microglia they stimulate the production and release of cytokines such as interleukins (IL). These interleukins are detected by astrocytes and neurons, where they stimulate signaling pathways that interfere directly with the signaling pathways activated during synaptic plasticity, inducing deficits in LTP or exacerbated LTD. Aβ peptides can also interfere directly with neurotransmitter receptors (Glutamate receptors (GluN, mGlu) or acetylcholine receptors) leading to abnormal neurotransmission. (top scheme, cerebral structure inspired from [37])

AD is a chronic neurodegenerative disease characterized by progressive neuronal loss and cognitive decline. Oligomeric amyloid β (oAβ) is implicated in the pathogenesis of AD and disrupts synaptic plasticity through numerous mechanisms (Fig. 3) [127]. Inflammatory features including activation and proliferation of glia and expression of mediators such as IL-1, IL-6, and TNFα [133, 134] have been clearly detected in the brain, CSF and peripheral blood of AD patients. These molecules are linked to immune cell activation and strongly affect LTP, even though the relation between oAβ and inflammation remains unclear. Regardless, it is conceivable that the ‘early’ loss of hippocampal LTP observed in AD represents a downstream effect of the presence of both oAβ and ongoing neuroinflammation.

Among cytokines, TNFα and IL-1β have been shown to mediate the detrimental effects of oAβ on LTP. Indeed, suppression of LTP by oAβ was absent in mutant mice null for TNF receptor type 1 and was prevented by the monoclonal antibody infliximab, the TNF peptide antagonist, and thalidomide, the inhibitor of TNFα production [135]. Further, intracerebroventricular administration of interleukin 1 receptor antagonist (IL-1ra) rescued post-tetanic potentiation impairment following injection of oAβ peptide [136].

To further support a role for inflammation in AD, evidence from epidemiologic studies and clinical trials suggest that non-steroidal anti-inflammatory agents (NSAIDs) exert neuroprotection in AD. Accordingly, two selective COX-2 inhibitors were effective in preventing the disruption of LTP by synthetic soluble Aβ [137].

An emerging issue is how microglia, which physiologically control synapse function and plasticity, contribute to AD pathogenesis. Recent evidence suggests that failure of signaling required for maintaining a ‘resting’ microglial phenotype, likely important for preserving surveillance functions, might have profound consequences on synaptic activity. For instance, numerous studies have found that Aβ increases microglia activation and the release of cytokines which impair LTP, and that inhibiting microglia activation can prevent the block of LTP induction by Aβ (for a recent review see [138]). Further the loss of synapses, a hallmark feature of AD, has been linked to microglia phagocytic activity. A recent study found that synapse loss in the hippocampus in the early stages of a mouse model of AD, as well as following direct infusion of oAβ, was mediated by the complement cascade (C1q, C3 and CR3). Thus the authors suggest that microglia-mediated synapse loss early in AD may be due to a pathological reactivation of a developmental program of synaptic pruning [139].

### Parkinson’s disease

Similar to the synaptotoxic role played by oAβ in AD, extracellular alpha-synuclein oligomers also modulate synaptic transmission and impair LTP [140]. However, results obtained in this study are questionable since alpha-synuclein oligomers were applied at supraphysiological concentrations. Aggregation of α-synuclein triggers the release of TNFα and IL-1β from microglia, and this might lead to its toxic effects on dopaminergic cells [141]. Several authors found elevated TNF, IL-1, IL-6, IL-2 and upregulation of MHC molecules in the striatum and CSF of PD patients [142], thus supporting the hypothesis that immune response is a pathogenic mechanism underlying PD. Notably, treatment with the flavonoid baicalein decreased upregulation of TNFα and IL-1β and normalized striatal glutamatergic transmission in a rodent model of PD [143]. Presence of a persistent active inflammatory process in PD patients might contribute to the impairment of physiological synaptic plasticity at corticostriatal synapses. This, in turn, might lead to disruption of signaling pathways within the basal ganglia neuronal network [144] as the basis of PD symptomatology.

Further studies are still required to clarify the precise role that cytokines might play in striatal synaptic plasticity in physiological and pathological conditions.

## Therapeutic implications and conclusions

During the course of an immune attack, release of pro-inflammatory cytokines is temporary and normally controlled by anti-inflammatory mechanisms, representing an adaptive and regulated response of the brain to immune signals. Conversely, when the immune challenge becomes prolonged and/or uncontrolled, the consequent inflammatory response might lead to pathological conditions.

Despite remarkable progress in the knowledge of cell signaling in neuroimmunology, to date several key questions still need to be addressed. For example, whereas immune protein function has been well characterized within the immune cells, not as much is known about how immune proteins exert their non-immune role to influence signaling pathways and gene expression engaged in synaptic plasticity in neurons. Next, it is still unclear how pathways are precisely activated by cytokines in target cells within a physiological or pathological setting. One critical issue might relate to the different concentrations of cytokines between the in vitro and in vivo condition. In fact, cytokines are generally used within the nanomolar range in vitro, whereas their in vivo levels in the brain fall within the picomolar range, making it difficult to define the realistic cytokine exposure at the synaptic level following different stimuli.

Considering the importance of immune mechanisms on neurotransmitter systems and brain circuitries relevant to neuropsychiatric diseases, a better understanding of brain-immune system interactions will hopefully provide specific biomarkers to measure the status of the neuroimmunological response, as well as novel neuroimmune-targeted therapeutics.

## Abbreviations

AD: Alzheimer’s disease
AMPAR: AMPA receptor
APP: Amyloid precursor protein
CaMKII: Ca2+/ calmodulin-dependent kinase II
CNS: Central nervous system
COX-2: Cyclooxygenase 2
cTBS: Continuous theta burst stimulations
DAMPs: Damage associated molecular patterns
dLGN: Dorsal lateral geniculate nucleus
EAE: Experimental autoimmune encephalomyelitis
eEF2K: Eucariotic elongation factor 2 kinase
GluA: Glutamate AMPA
GluK: Glutamate kainate
GluN: Glutamate NMDA
GluRs: Glutamate receptors
GPCRs: G protein coupled receptors
IFNα: Interferon α
IFNγ: Interferon γ
IL-18: Interleukin 18
IL-1β: Interleukin 1β
IL-2: Interleukin 2
IL-6: Interleukin 6
IL-8: Interleukin 8
iTBS: Intermittent theta burst stimulations
LTD: Long term depression
LTP: Long term potentiation
MD: Monocular deprivation
mEPSC: Miniature excitatory post synaptic current
mGlu: Metabotropic glutamate
MHC: Major histocompatibility complex
MS: Multiple sclerosis
NMDAR: NMDA receptor
NSAIDs: Non-steroidal anti-inflammatory agents
oAβ: Oligomeric amyloid β
PAMPs: Pathogen associated molecular patterns
PD: Parkinson’s disease
PI3K: Phosphoinositide-3-kinase
PKA: Protein kinase A
PKC: Protein kinase C
PLC: Phospholipase C
PRR: Pattern recognition receptors
RGCs: Retinal ganglion cells
STD: Short term depression
STP: Short term potentiation
TBI: Traumatic brain injury
TLRs: Toll-like receptors
TNF: Tumor necrosis factor
TNFα: Tumor necrosis factor α
TTX: Tetrodotoxin
WNV: West Nile virus
